# Instant Controlled Pressure Drop as Blanching and Texturing Pre-Treatment to Preserve the Antioxidant Compounds of Red Dried Beetroot (*Beta vulgaris* L.)

**DOI:** 10.3390/molecules25184132

**Published:** 2020-09-10

**Authors:** Maritza Alonzo-Macías, Anaberta Cardador-Martínez, Colette Besombes, Karim Allaf, Viridiana Tejada-Ortigoza, Marla C. Soria-Mejía, Rosa Vázquez-García, Carmen Téllez-Pérez

**Affiliations:** 1 Tecnologico de Monterrey, Escuela de Ingeniería y Ciencias, Epigmenio González 500, Fracc. San Pablo, 76130 Querétaro, Mexico; malonzoma@tec.mx (M.A.-M.); mcardador@tec.mx (A.C.-M.); viri.tejada@tec.mx (V.T.-O.); marlasoriamejia@gmail.com (M.C.S.-M.); rosavazquezg7@gmail.com (R.V.-G.); 2 Intensification of Transfer Phenomena on Industrial Eco-Processes, Laboratory of Engineering Science for Environment LaSIE-UMR-CNRS 7356, University of La Rochelle, 17042 La Rochelle, France; colette.besombes@univ-lr.fr (C.B.); kallaf@univ-lr.fr (K.A.)

**Keywords:** red beetroot, *Beta vulgaris* L., drying, blanching, phenolics, antioxidant activity, swell drying

## Abstract

Red beetroot is rich in bioactive compounds such as polyphenols, flavonoids, betaxanthins, betacyanins, among others. According to selected processing methods, the bioaccessibility of these compounds could be either enhanced or decreased. This study evaluated the effect of four different drying conditions: (1) Traditional Drying (TD), (2) Swell Drying (SD), (3) DIC Blanching + Traditional Drying (BTD), and (4) DIC Blanching + Swell Drying (BSD) on the antioxidant content and the antioxidant activity of red beetroots. Obtained results showed that in all the cases, by comparing to Traditional Drying (TD), the coupling of a DIC Blanching pre-treatment to a Swell Drying treatment (BSD) maintained or enhanced the preservation of the Total Phenolic Compounds (TPC), the Total Flavonoids Compounds (TFC), the Betanin Concentration (BC), the Trolox Equivalent Antioxidant Capacity (TEAC), and the Free Radical Scavenging Activity by DPPH (IC50) of red beetroots. Various studies have shown that thanks to the expanded and porous structure triggered by the Swell Drying process, it has been possible to achieve better antioxidants extraction and better whole quality. Hence, by coupling DIC as a blanching–steaming pre-treatment, it was possible to preserve better the antioxidant content and the antioxidant activity of red dried beetroots.

## 1. Introduction

Red beetroot (*Beta vulgaris* L.) is known for its valuable active and health-promoting compounds like carotenoids, glycine betaine, saponins, betacyanins, folates, betanin, polyphenols, flavonoids, calcium, biotin, dietary fiber, among others [1,2,3]. Moreover, red beetroot pigments are of particular interest in the industry as safe color additives and nutraceuticals [4]. The main red-purple pigments in red beetroot are water-soluble betalains, a class of betalamic acid derivatives mainly composed by the red-violet betacyanins (400–2100 mg/kg fresh weight), like betanin, and by the yellow-orange betaxanthins (200–1400 mg/kg fresh weight), such as vulgaxanthin I. Betanin, or betanidin-5-*O*-β-glycoside, is shown to be the most abundant betalain in red beetroot, composing from 75–95% of the mass fraction of the vegetable [1,5,6,7]. Betalains are sensitive to degradation via heat, light, enzymes, and oxygen; thus, drying conditions are essential for their preservation [8]. Convective airflow drying is one of the most used preservation methods. However, the long periods and high temperatures that might be applied during this process lead to undesirable changes and the degradation of important phytochemicals, such as antioxidant compounds [3,5]. For example, the study of Guldiken, et al. [9] showed that oven-drying (80 °C/8 h, with a final moisture content of 50% on wet basis—w.b.) of fresh red beetroot caused 64% and 77% losses of Total Phenolics Content and Total Flavonoids Content; and 74% and 75% losses of Total Antioxidant Capacity by TEAC and DPPH, respectively. As a response, novel drying technologies have been applied to improve the preservation of natural food compounds.

Swell-drying (SD) is an innovative drying process that couples the benefits of convective airflow drying to the Instant Controlled Pressure Drop (DIC) technology. DIC, by its French acronym “Détente Instantanée Contrôlée,” is a high temperature/short time (HTST) process. It consists of subjecting a food material to high pressure and high-temperature treatments (from 0.1 up to 1 MPa) followed with an abrupt pressure drop (ΔP/Δt > 0.5 MPa s^−1^) towards a vacuum (around 4.5 kPa). After the pressure drops, several thermo-mechanical effects occur into the food material, such as expansion, instantaneous cooling of the products, and the rapid vaporization of volatile compounds [10]. Many fruit and vegetable materials have been subjected to this technology, such as strawberry [11,12], banana [13], pepper [14,15], garambullo [16], date [17] and chokecherry [18]; and research findings have shown that thanks to a porous structure newly generated by DIC, swell-drying reduced the drying time and preserved bioactive and nutritional food compounds.

On the other hand, DIC has also been applied as a blanching–steaming pre-treatment before convective air drying, and the results have shown that when DIC is applied to fresh food material, it reduced the drying time, which triggered better preservation of nutritional and sensorial attributes of dried products. In the case of onion, by comparing the drying performance of DIC blanching samples to raw material, DIC achieved a reduction of drying time around 78% [19].

Therefore, this work aimed to determine DIC technology’s effect as blanching and texturing pre-treatment on the antioxidant content and antioxidant activity preservation of red dried beetroots. A central composite rotatable design was applied to evaluate the DIC technology operating parameters (steam pressure and thermal processing time) on the Total Phenolic Compounds (TPC), the Total Flavonoids Compounds (TFC), the Betanin Concentration (BC), the Trolox Equivalent Antioxidant Capacity (TEAC), and the Free Radical Scavenging Activity by DPPH (IC_50_).

## 2. Results

### 2.1. Proximal Analysis

Proximate analysis of red dried beetroots (g/100 g of dry solids) presented crude protein (nitrogen × 6.25) of 1.08 ± 0.11 g, total lipids of 1.43 ± 0.27 g, ash of 6.20 ± 0.23 g, crude fiber of 4.51 ± 0.03, and total carbohydrates (calculated by difference) of 86.78 ± 0.19 g. The raw material’s initial moisture content was 85.90% ± 0.56 w.b., and the final moisture content of all dried red beetroots was 4.09% ± 0.37 w.b.

### 2.2. Bioactive Compounds Quantification

**Figure 1:**
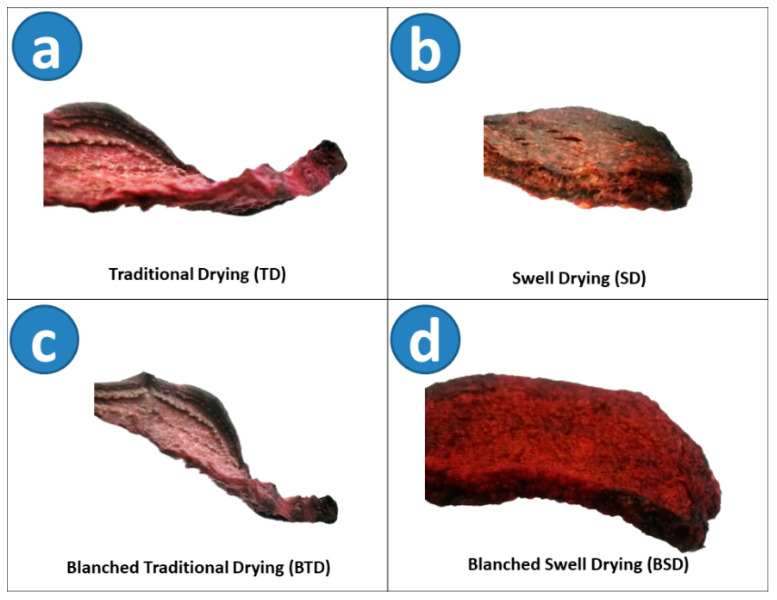
Photographs of (**a**) Traditional Drying (TD), (**b**) Swell Drying (SD), (**c**) Blanched Traditional Drying (BTD), and (**d**) Blanched Swell Drying (BSD) of red beetroots.

Table 1 shows the obtained results of Total Phenol Content (TPC), Total Flavonoids Content (TFC), and Betanin Concentration (BC) of all dried red beetroots. Figure 1 shows the appearance of (a) Traditional Drying (TD), (b) Swell Drying (SD), (c) DIC Blanching + Traditional Drying (BTD), and (d) DIC Blanching + Swell Drying (BSD) of red beetroots.

#### 2.2.1. Total Phenol Content

**Figure 2:**
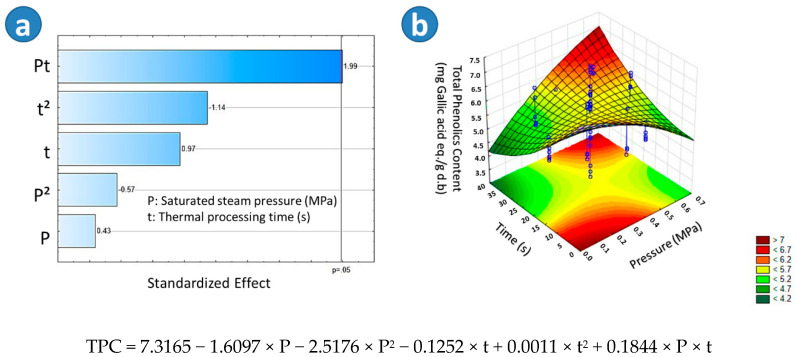
Effects of steam pressure “P” (MPa) and thermal processing time “t” (s) on the Total Phenol content (TPC) of Blanched Swell-Dried red beetroots (BSD). (**a**) The Pareto chart and (**b**) surface response plot are shown.

As shown in Table 1, BSD13 presented the highest TPC (7.03 mg Gallic acid eq./g d.b.), followed by TD (6.95 mg Gallic acid eq./g d.b.), BTD (6.62 mg Gallic acid eq./g d.b.) and SD (6.59 mg Gallic acid eq./g d.b.). According to the Tukey test, there was no significant difference in the TPC between SD and BTD. Moreover, regarding the Pareto chart in Figure 2a, it could be observed that in the case of blanched swell-dried samples, the interaction of the saturated steam pressure and the thermal processing time (Pt) had a significant effect on increasing the TPC. On the other hand, Figure 2b showed that the higher the “P” and “t”, the higher the TPC of BSD samples. Adequate empirical regression model of the TPC of blanched swell dried red beetroots is shown in Figure 2.

#### 2.2.2. Total Flavonoid Content

**Figure 3:**
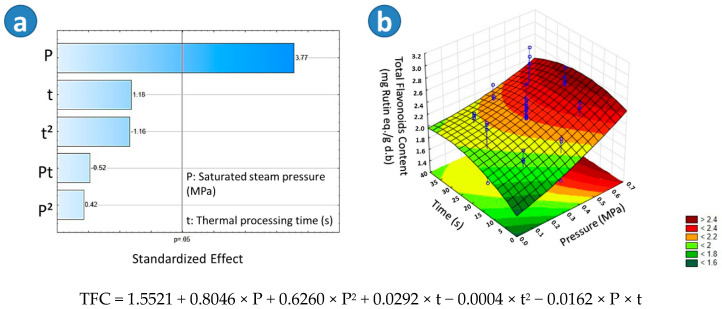
Effects of steam pressure “P” (MPa) and thermal processing time “t” (s) on the Total Flavonoids Content (TFC) of Blanched Swell-Dried (BSD) red beetroots. (**a**) The Pareto chart and (**b**) surface response plot are shown.

The highest Total Flavonoids Content was found in the sample BSD2 (2.54 mg rutin eq./g d.b), followed by SD (2.42 mg rutin eq./g d.b). With respect to BTD (2.13 mg rutin eq./g d.b) and TD (2.04 mg rutin eq./g d.b) samples, a slight decrease on TFC could be observed. According to the Tukey test, there was a significant difference in the TFC between BTD and TD. On the other hand, regarding the Pareto chart in Figure 3a, it could be observed that in the case of blanched swell-dried samples, the saturated steam pressure (P) had a significant effect on the TFC. Figure 3b showed that the higher the “P,” the higher the TFC of BSD samples. An adequate empirical regression model of the TFC of blanched swell-dried red beetroots is shown in Figure 3.

#### 2.2.3. Betanin Concentration

**Figure 4:**
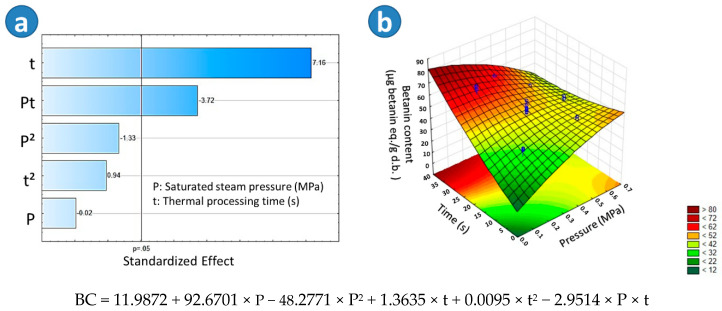
Effects of steam pressure “P” (MPa) and thermal processing time “t” (s) on the Betanin Concentration (BC) of Blanched Swell-Dried (BSD) red beetroots. (**a**) The Pareto chart and (**b**) surface response plot are shown.

The highest betanin concentration was found in the sample BSD9 (67.50 μg betanin eq./g d.b). By comparing this result to TD (49.36 μg betanin eq./g d.b), BTD (40.92 μg betanin eq./g d.b), and SD (30.56 μg betanin eq./g d.b), a reduction of betanin concentration by TD, BTD, and SD could be observed. In fact, according to the Tukey test, there was a significant difference in the betanin concentration among BSD9, TD, BTD, and SD, the lowest betanin concentration being found in the SD beetroots. By regarding the Pareto chart in Figure 4a, it could be observed that in the case of blanched swell-dried samples, the thermal processing time (t) and the interaction of the saturated steam pressure and the thermal processing time (Pt) had a significant effect on the Betanin Concentration (BC). Figure 4b showed that the higher the “t” and the lower the “P”, the higher the BC of BSD samples. An adequate empirical regression model of the BC of blanched swell-dried red beetroots is shown in Figure 4.

### 2.3. Antioxidant Activity

Table 2 shows the obtained results of Trolox Equivalent Antioxidant Capacity (TEAC) and Free Radical Scavenging Activity by DPPH (IC_50_) of all dried red beetroots.

#### 2.3.1. Trolox Equivalent Antioxidant Capacity (TEAC)

**Figure 5:**
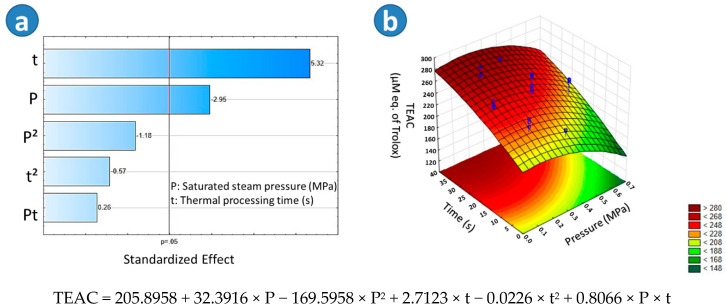
Effects of steam pressure “P” (MPa) and thermal processing time “t” (s) on the Trolox equivalent antioxidant capacity (TEAC) of Blanched Swell-Dried (BSD) red beetroots. (**a**) The Pareto chart and (**b**) surface response plot are shown.

The samples that presented the higher TEAC values were the BSD4 (305.67 μM eq. of Trolox), BSD3 (298.72 μM eq. of Trolox), and TD samples (278.03 μM eq. of Trolox). With respect to SD (202.06 μM eq. of Trolox) and BTD (182.19 μM eq. of Trolox), it could be observed that both drying treatments decreased the TEAC of beetroots. According to the Tukey test, there was a significant difference among TD, BTD, and SD, the lowest TEAC being found in BTD beetroots. Regarding the Pareto chart in Figure 5a, it could be observed that in the case of blanched swell-dried samples, the thermal processing time (t) and the saturated steam pressure (P) had a significant effect on the TEAC. Figure 5b showed that the higher the “t” and the lower the “P”, the higher the TEAC of BSD samples. An adequate empirical regression model of the TEAC of blanched swell dried red beetroots is shown in Figure 5.

#### 2.3.2. Free Radical Scavenging Activity by DPPH (IC_50_)

**Figure 6:**
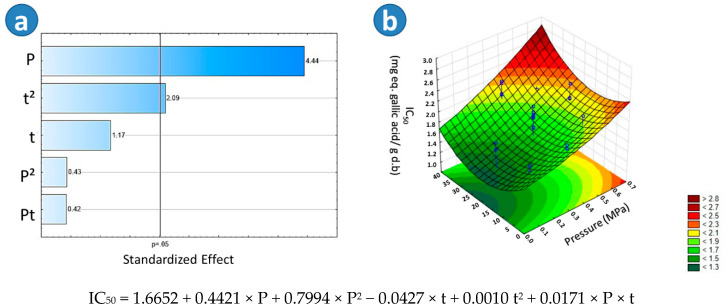
The effects of steam pressure “P” (MPa) and thermal processing time “t” (s) on the free radical scavenging activity by DPPH (IC_50_) of Blanched Swell-Dried (BSD) red beetroots. (**a**) The Pareto chart and (**b**) surface response plot are shown.

As shown on Table 2, the samples BSD10 (0.84 mg eq. gallic acid/g d.b) and BSD11 (0.99 mg eq. gallic acid/g d.b) showed the lower concentrations to decolorate 50% of DPPH (IC_50_), which means a higher free radical scavenging activity. With respect to SD (1.38 mg eq. gallic acid/g d.b) and BTD (1.40 mg eq. gallic acid/g d.b), it could be observed that those drying treatments decreased the free radical scavenging activity of red beetroots. According to the Tukey test, there was not a significant difference between them. Among all studied drying treatments, TD samples (2.69 mg eq. gallic acid/g d.b) presented the lowest free radical scavenging activity. By regarding the Pareto chart in Figure 6a, it could be observed that in the case of blanched swell-dried samples, the saturated steam pressure (P) and the quadratic effect of the thermal processing time (t^2^) had a significant impact on the IC_50_. Figure 6b showed that the lower the “P”, the lower the IC_50_ of BSD samples. An adequate empirical regression model of the IC_50_ of blanched swell-dried red beetroots is shown in Figure 6.

## 3. Discussion

### 3.1. Proximal Analysis

By comparing our results to the study of Nistor, et al. [20], it could be observed that both fresh red beetroot samples presented almost the same initial moisture content (85.90% w.b. vs. 86.26% w.b.). Moreover, a similar behavior was found in the ash content (6.20 g/100 dry solids vs. 6.81 g/100 dry solids) and in total lipid content (1.43 g/100 dry solids vs. 1.02 g/100 dry solids). Regarding the carbohydrates content (86.78 g/100 dry solids vs. 64.16 g/100 dry solids), our red beetroot samples presented higher values, but lower crude protein content (1.08 g/100 dry solids vs. 10.83 g/100 dry solids) and crude fiber content (4.51 g/100 dry solids vs. 17.17 g/100 dry solids). The variability of red beetroot composition between both samples could be linked to various factors, such as the ripeness stage at harvesting, the growing location, and the environmental conditions, among others.

### 3.2. Bioactive Compounds Quantification

Red beetroot is rich in bioactive compounds as polyphenols, flavonoids, betalains, among others. Hence, according to selected processing methods, the bioaccessibility of these compounds could be either enhanced or decreased. This study evaluated the effects of four different drying conditions (TD, SD, BTD, and BSD) of red beetroots on their Total Phenol Content (TPC), their Total Flavonoids Content (TFC), and their Betanin Concentration (BC). Results showed that, in all the cases, when compared to Traditional Drying (TD), the coupling of a DIC Blanching pre-treatment with a Swell Drying treatment (BSD) maintained or enhanced the preservation of TPC (1% higher), TFC (25% higher), and BC (37% higher). Blanching is an essential step in fruit and vegetable processing commonly employed to inactivate deleterious enzymes and to reduce the microbial load. However, if it is not accurately applied, it could trigger adverse effects, such as pigment modifications and nutrient losses [21]. DIC as a blanching–steaming pre-treatment may help to inactivate peroxidase and polyphenoloxidase, both considered heat resistance enzymes. Moreover, as DIC Blanching pre-treatment involves an instant cooling of food samples at the pressure drop stage, it may stop the thermal degradation of bioactive compounds.

On the other hand, even if well-controlled blanching pre-treatment contributes to preserving food phytochemicals, the adequate selection of further processing is also vital. In this context, by coupling swell drying with previous DIC blanched red beetroots, it was possible to obtain a more porous structure that may allow better extraction of bioactive compounds. As shown in Figure 1, TD and BTD performed the most compact structure, not the same for SD and BSD, which presented a more porous structure.

On the other hand, in the case of BTD and SD, the slight loss of TPC respect to TD (~5% in both cases) could be due to the thermal deterioration of the phenolics during drying. Moreover, as TPC was determined by the Folin-Ciocalteu method, it is important to consider that it measures a sample’s reducing capacity. Then, it also could quantify other compounds, like vitamin C, amino acids, peptides, organic acids, and Maillard reaction products. Additionally, some of them are more sensitive to temperature, like vitamin C [22,23].

Regarding the TFC results, it could be observed that compared to TD, BTD and SD enhanced the TFC into 4% and 19%, respectively. Because flavonoids are thermolabile compounds, losses of TFC by TD could be linked to long convective hot air drying. On the other side, regarding the betanin concentration (BC) results, it can be highlighted that TD, BTD, and SD provoked losses of BC of 17% and 38%, respectively. This decreasing trend could be attributed to the fact that betanin is very sensitive to degradation by heat, enzymes, and oxygen [6]. Additionally, Nistor, Seremet, Andronoiu, Rudi and Botez [20] described that convective drying induces betalains’ oxidation due to prolonged exposure of the samples to thermal treatment. Besides, Ravichandran, et al. [24] indicated that betalains from dried beetroots are more stable than in raw products, because of the reduced water content, the enzymatic inactivation, and the impermeability of the dried products. On the other hand, the differences between BTD and SD may be linked to a better inactivation of oxidative enzymes (e.g., peroxidase) [8], by DIC blanching, which probably reduced the betanin oxidation.

### 3.3. Antioxidant Activity

Various studies have shown that fresh red beetroots posess high antioxidant activity [25,26,27]. However, an inadequate selection of processing methods may have harmful effects on their antioxidant potential. In this sense, this study showed that, according to the selected conditions of beetroot drying, the antioxidant capacity could vary significantly. Regarding the Trolox Equivalent Antioxidant Capacity (TEAC) and the Free Radical Scavenging Activity by DPPH (IC_50_) of the four studied drying treatments (TD, BTD, SD, and BSD), it could be highlighted that, in both cases, the highest antioxidant activities were performed by BSD samples (DIC blanching of 0.30 MPa and 20 s + pre-drying + swell drying texturization of 0.35 MPa and 20 s). These results could be linked to better preservation of the antioxidant compounds by DIC blanching pre-treatment, and better extraction of antioxidants attributed to the new porous structure triggered by swell drying. With respect to TD, BSD samples enhanced the TEAC by 10%, and the IC_50_ by 3.2 times.

By regarding the TEAC of BTD and SD with respect to TD, it can be remarked that both reduced by 34% and 27%, respectively, the antioxidant activity of beetroot samples. These results agree with the losses of phenolics compounds and betanin concentration of both samples. Moreover, even if SD presented the highest losses of betanin concentration, at the same time, this treatment preserved better the flavonoid compounds. Then, it could be possible that flavonoids in beetroots exerted better TEAC. On the other hand, it could also be possible that other bioactive compounds, such as betaxanthins and vitamins, also performed effective antioxidant properties [28].

In the case of IC_50_, BTD and SD increased in 1.92 and 1.95 times, respectively, the antioxidant activity of beetroot samples by comparing them to TD. As shown by the study of Abramovič, et al. [29], even if TEAC and IC_50_ measure the number of hydrogen/electrons that an extract can exchange with the oxidant probe, the overall reaction rate depends on the proportion of ionized molecules of the antioxidant, which implies the influence of experimental parameters. Then, the divergences between TEAC and IC_50_ results could be attributed to the differences in the chemical structures of the antioxidant molecules, the pH of reaction media, the length of the assay, and the solvent composition, among others [29].

Finally, to attain a better understanding of the changes produced by the DIC blanching and texturing treatment on the antioxidant content of red dried beetroots, it is suggested to optimize the extraction of bioactive compounds to analyze the phenolics and flavonoids composition via HPLC and to evaluate the inactivation of oxidative enzymes via peroxidase and polyphenol oxidase assays.

## 4. Materials and Methods

### 4.1. Materials

#### 4.1.1. Biological Material

Organic raw red beetroots (*Beta vulgaris* L.) were bought in a local market in La Rochelle, France. They were stored at the laboratory for 24 h at room temperature (~23 °C).

#### 4.1.2. Reagents and Solvents

Gallic acid, rutin, betanin, 1,6-hydroxy-2,5,7,8-tetramethylchroman-2-carboxylic acid (Trolox), potassium persulfate, Folin–Ciocalteu reagent, 2,2-diphenyl-1-picrylhydrazyl (DPPH) and 2,2′-azino-bis (3-ethylbenzothiazoline)-6-sulfonic acid (ABTS) were purchased from Sigma-Aldrich Canada Ltd. (Oakville, ON, Canada). HPLC grade methanol was obtained from Karal (León; Guanajuato, Mexico). The water used was obtained from a Millipore Milli-Q water system with a resistivity of 18.2 MΩ cm (25 °C).

### 4.2. Methods

#### 4.2.1. Blanching and Drying Procedures

Before any treatments, good quality red beetroots were washed, peeled, and sliced to an average thickness of 1/4 inches. To evaluate the effects of the Instant Controlled Pressure Drop as blanching and texturing pre-treatment, fresh red beetroots with an initial moisture content of 85.90% wet basis (w.b.), were divided into three groups:

(1) The raw material for Traditional Drying (TD)
(2) The raw material for Swell Drying (SD)
(3) The raw material for DIC Blanching (B)

Moreover, to assess the impact of coupling DIC blanching to traditional drying and swell drying, DIC blanched beetroots were subdivided into two groups:

(4) DIC Blanching + Traditional Drying (BTD)
(5) DIC Blanching + Swell Drying (BSD)

**Figure 7:**
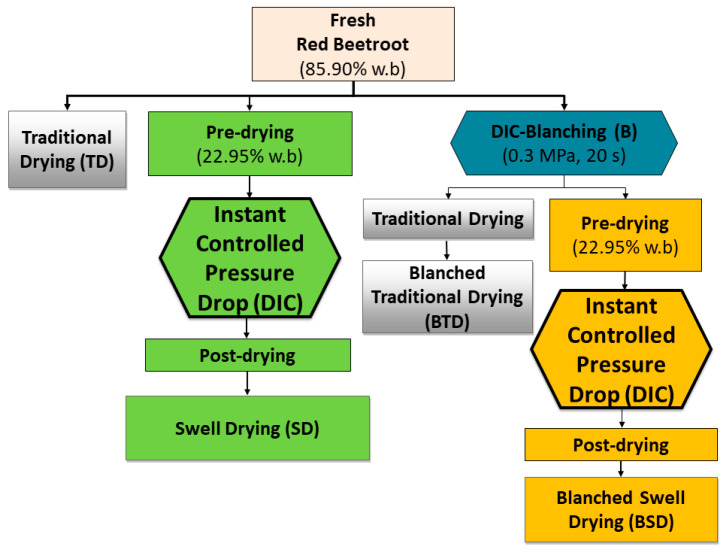
Schematic diagram of blanching, texturing, and drying treatments applied to red beetroots.

In all drying steps (traditional drying, pre-drying, and post-drying), a laboratory drying oven UFE 400 (Memmert, Schwabach, Germany) was used. Convective air-drying conditions were 60 °C of temperature, 265 Pa of the partial pressure of vapor, and 1.2 ms^−1^ of airflow velocity. Figure 7 shows a schematic diagram of blanching, texturing, and drying treatments applied to red beetroots.

Traditional Drying (TD) consisted of drying fresh red beetroots until reaching a ~4% w.b. moisture content. Swell Drying (SD) consisted of three processing stages:

(a) A pre-drying of red beetroots via convective air drying until reaching a moisture content of 22.95% w.b.
(b) A DIC treatment (saturated steam pressure “P” of 0.35 MPa and thermal treatment time “t” of 20 s)
(c) A post-drying via convective air drying until reaching a ~4% w.b. moisture content.

DIC blanching (B) consisted of submitted fresh red beetroots to a DIC treatment under a P = 0.3 MPa and a t = 20 s. The Blanched Traditional Drying (BTD) treatment consisted of drying DIC blanched red beetroots until reaching a ~4% w.b. moisture content. Blanched Swell Drying (BSD) treatment consisted of pre-drying DIC blanched beetroots until a moisture content of 22.95% w.b., followed by DIC treatments under a five-level central composite rotatable design and a final post-drying until a ~4% w.b. moisture content. All dried samples (TD, SD, BTD, and BSD) were packed in airtight bags and stored at room temperature until further assessments. In all cases, the DIC treatment was carried on the laboratory DIC MP reactor (manufactured at ABCAR-DIC Process; La Rochelle; France).

**Figure 8:**
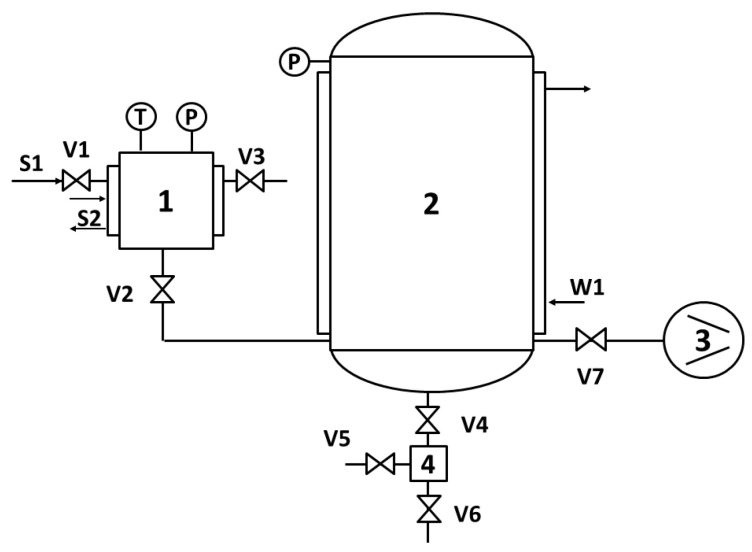
Schematic diagram of DIC Equipment (ABCAR-DIC Process; La Rochelle; France): (1) DIC reactor, (2) vacuum tank, (3) vacuum pump, (4) trap, V1-V7-valves, S1, and S2—saturated steam injection; W1—cooling water, P—pressure gauge and T—thermocouples.

The DIC equipment MP reactor consisted of three major components: (i) a double jacket processing vessel, (ii) a vacuum system, and (iii) the decompression system [30]. Figure 8 shows the schematic diagram of the DIC equipment reactor.

**Figure 9:**
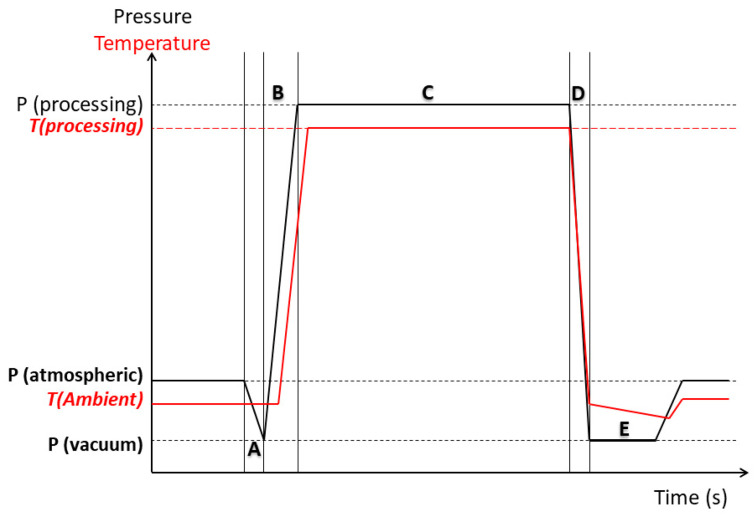
Schematic time-pressure profiles of a DIC processing cycle. (A): The establishment of the vacuum; (B): the injection of steam; (C) maintained treatment pressure during the selected thermal processing time; (D): Instant Controlled Pressure Drop towards the vacuum; (E): the establishment of the atmospheric pressure.

For the DIC treatment of fresh and predried red beetroots, samples were introduced on the DIC processing reactor (Figure 8). Then, inside the reactor, a vacuum of approximately 5 kPa was established (Figure 9A). Next, saturated steam was injected into the reactor at a fixed pressure level (P) (Figure 9B), and once selected pressure was achieved, this was maintained for a given thermal processing time “t” (Figure 9C). Selected operating parameters of “P” and “t” were defined by preliminary studies. Once thermal processing time was finished, samples were subjected to an Instant Controlled Pressure Drop (ΔP/Δt > 0.5 MPa s^−1^) towards a vacuum of 5 kPa (Figure 9D). After a vacuum stage period time of 2 s, the pressure was released toward the atmospheric pressure (Figure 9E), and samples were recovered from the reactor.

#### 4.2.2. Experimental Design

A central composite rotatable design was applied to assess the effect of DIC operating parameters on BSD red beetroots. Steam pressure (P) and thermal processing time (t) were the independent variables. The design yielded 13 experiments with four factorial points (2^2^), four-star points (−α, −1, +1, and +α), and five central points (0,0). The experimental design was run randomly to minimize the effects of unexpected variability on observed responses due to external factors. In each run, 40 g of blanched predried red beetroots were used. Table 3 shows the experimental design for BSD red beetroots.

#### 4.2.3. Proximal Analysis

Moisture (930.15), protein (981.10), fat (920.39), ash (942.05), and crude fiber (962.09) were analyzed according to the AOAC Official Methods [31].

#### 4.2.4. Bioactive Compounds Quantification and Antioxidant Activity

To evaluate the effect of the Instant Controlled Pressure Drop as blanching and texturing pre-treatment on the antioxidant content and the antioxidant activity of red dried beetroot, the Total Phenolics Content (TPC), the Total Flavonoids Content (TFC), the Betanin Concentration (BC), the Trolox Equivalent Antioxidant Capacity assay (TEAC), and the Free Radical Scavenging Activity by DPPH (IC_50_) where evaluated.

### 4.3. Methanolic Extracts Preparation

Before extraction, dried red beetroots samples were milled and sieved through 60 mesh. Afterward, 0.5 g of beetroots were extracted with 10 mL of methanol with 1% of HCl (0.1 M). The extraction was carried out in the absence of light, at room temperature at 25 °C, and under agitation for two hours. Sample suspensions were centrifuged for 10 min at 4 °C and 6000 rpm (Hermle Z 383 K, Wehingen, Germany). Extracts were prepared by duplicate, and supernatants were stored at −20 °C until analysis.

### 4.4. Total Phenolics Content

The Folin-Ciocalteu colorimetric method adapted to a 96-well microplate [32] was used to determined Total Phenolic Content (TPC). Briefly, 0.02 mL of the extract was oxidized with 0.1 mL of 0.5 N Folin-Ciocalteu reagent, and after 5–8 min, the reaction was neutralized with 0.3 mL sodium carbonate solution (20%). The absorbance values were obtained with a Spectrophotometer (xMark Microplate Spectrophotometer, BioRad, Osaka, Japan) by measuring the resulting blue color at 760 nm after incubation for 2 h at 25 °C. A standard curve of gallic acid ranging from 0 to 500 μg/mL was done to quantify the TPC. Results were expressed as mg of gallic acid equivalent per 100 g of dry matter (mg gallic acid eq./g d.b.).

### 4.5. Total Flavonoids Content

Total Flavonoid Content (TFC) was determined, according to Oomah, et al. [33]. The method consisted of mixing in a 96-well microtitration flat-bottom plate, 50 μL of the methanolic extract with 180 μL of distilled water, and 20 μL of a solution of 10 g/L 2-aminoethyldiphenylborate. The absorbance of the solution was measured with a spectrophotometer (xMark Microplate Spectrophotometer, BioRad, Osaka, Japan) at 404 nm after 15 min of reaction. Extract absorption was compared with a rutin standard (0 to 200 μg/mL). TFC was expressed as mg rutin equivalent per 100 g of dry matter (mg eq. rutin/g db).

### 4.6. Betanin Content by HPLC

Red beetroot methanol sample extracts were filtered through a 0.45 μm nylon membrane filter and injected into an Agilent 1200 HPLC system (Agilent Technology 1200 series, Palo Alto, CA, USA) equipped with an Agilent Eclipse XDB-C18 column (5 μm, 4.6 mm, 150 mm) at 28 °C. The mobile phase was 0.1% (*v*/*v*) trifluoroacetic acid/water (A) and acetonitrile with 0.1% (*v*/*v*) trifluoroacetic acid B). The flow rate was 1 mL/min. The linear gradient conditions were 95% solvent A from 0 to 45 min; followed by 65% solvent A from 45 to 47 min; continued by 25% solvent from 47 to 54 min, and at 54 min returned to the initial conditions [9]. The diode array detector was set at 535 nm. Betanidin 5-*O*-β-glycoside (betanin) was used as a standard (0–80 μg/mL). The betanin concentration was expressed as μg betanin equivalent per g of dry matter (μg betanin eq./g of d.b.).

### 4.7. Trolox Equivalent Antioxidant Capacity (TEAC)

The Trolox equivalent antioxidant capacity assay (TEAC) was performed according to Re, et al. [34]. First, the ABTS radical cation (ABTS•+) was produced by mixing ABTS (7 mM) with potassium persulphate (2.45 mM), and the reaction mixture was stored at room temperature in the dark for 12 to 16 h. After that, the ABTS•+ stock solution was diluted with ethanol to an absorbance of 0.8 ± 0.1 at 734 nm. Subsequently, 0.02 mL of sample or Trolox standard (from 0 to 500 μM) were added in a 96-microplate, followed by 0.2 mL of ABTS•+ reagent, and they were mixed thoroughly. The absorbance readings were taken after 6 min at 734 nm by using a visible-UV microplate reader (xMark Microplate Spectrophotometer, BioRad). The TEAC of samples was expressed as the μM of Trolox needed to give the same degree of discoloration as the samples (μM eq. of Trolox). Each sample was analyzed in triplicate.

### 4.8. Free Radical Scavenging Activity by DPPH (IC_50_)

Free radical scavenging activity was determined by the method of Fukumoto and Mazza [35] and Burda and Oleszek [36]. The 2,2-diphenyl-1-picrylhydrazyl (DPPH) was used as a free radical. 20 μL of the extract at three different concentrations were mixed with 200 μL DPPH (125 μM in 80% methanol) in a 96-well microtitration flat-bottom plate. Then, after 90 min, the plate was read at 520 nm in a spectrophotometer (xMark Microplate Spectrophotometer, BioRad). The free radical scavenging concentration necessary to decolorate 50% of DPPH (IC_50_) was calculated by linear regression and expressed as mg eq. Gallic acid/g of dry matter. The analysis was performed in triplicate.

### 4.9. Statistical Analysis

The Statistica Software 2017 (TIBCO Software Inc., Palo Alto, California, CA, USA) was used for the statistical analysis. To evaluate any significant difference between each treatment, the analysis of variance (ANOVA) (*p* < 0.05) and the multiple comparisons by Tukey’s honest significant test (α < 0.05) were applied. For the experimental design of DIC treatment, statistical analysis was performed through the Pareto chart and the surface response methodology. The Pareto chart was used to identify the impact of variables on the responses. The vertical line in the Pareto chart determines the statistically significant effects at the 95% confidence level. Saturated steam pressure (MPa) and thermal processing time (s) were studied as independent variables. The studied response variables were: Total Phenolics Content (TPC), Total Flavonoids Content (TFC), Betanin Concentration (BC) and Antioxidant Activity (TEAC and IC_50_).

## 5. Conclusions

This study focused on evaluating the effects of four drying conditions: (1) Traditional Drying (TD), (2) Swell Drying (SD), (3) DIC Blanching + Traditional Drying (BTD), and (4) DIC Blanching + Swell Drying (BSD), on the antioxidant content and the antioxidant activity preservation of red beetroots. The results showed that the coupling of a DIC Blanching pre-treatment with a Swell Drying treatment (BSD) maintained or enhanced the preservation of the Total Phenolic Compounds (TPC), the Total Flavonoids Compounds (TFC), the Betanin Concentration (BC), the Trolox Equivalent Antioxidant Capacity (TEAC), and the Free Radical Scavenging Activity by DPPH (IC_50_) of red dried beetroots. Then, by coupling DIC as a blanching-steaming pre-treatment with swell drying as a texturing treatment, it was possible to preserve the antioxidant compounds of red dried beetroots.

## Figures and Tables

**Table 1:** Total Phenolics Content (TPC), Total Flavonoids Content (TFC), and Betanin Concentration (BC) of red dried beetroots.

Treatment	Total Phenolics Content (TPC) ^1^	Total Flavonoids Content (TFC) ^2^	Betanin Concentration (BC) ^3^
TD	6.95 ± 0.52 ^a,b^	2.04 ± 0.11 ^f,g^	49.36 ± 0.90 ^c,d^
SD	6.59 ± 0.74 ^a,b,c^	2.42 ± 0.10 ^a,b^	30.56 ± 0.18 ^h^
BTD	6.62 ± 1.20 ^a,b,c^	2.13 ± 0.14 ^c,d,e^	40.92 ± 2.86 ^f^
BSD1	6.21 ± 0.52 ^b,c,d,e^	2.38 ± 0.18 ^a,b,c^	48.71 ± 2.46 ^d,e^
BSD2	6.01 ± 0.63 ^c,d,e^	2.54 ± 0.15 ^a^	48.67 ± 1.39 ^d,e^
BSD3	5.48 ± 0.18 ^d,e,f^	2.15 ± 0.18 ^c,d,e^	59.40 ± 3.92 ^b^
BSD4	5.31 ± 0.11 ^e,f,g^	2.25 ± 0.18 ^c,d,e^	51.12 ± 0.21 ^c^
BSD5	5.95 ± 0.27 ^c,d,e,f^	2.30 ± 0.56 ^b,c,d^	49.27 ± 0.82 ^c,d^
BSD6	5.17 ± 0.23 ^e,f,g^	2.22 ± 0.25 ^c,d,e^	45.70 ± 1.72 ^e^
BSD7	5.73 ± 0.45 ^d,e,f^	2.11 ± 0.19 ^d,e,f^	51.09 ± 1.47 ^c^
BSD8	6.34 ± 1.12 ^b,c,d^	1.91 ± 0.24 ^g^	42.13 ± 0.50 ^f^
BSD9	5.66 ± 0.56 ^d,e,f^	2.11 ± 0.15 ^c,d,e^	67.50 ± 1.28 ^a^
BSD10	4.22 ± 0.65 ^h^	2.06 ± 0.53 ^e,f^	56.79 ± 3.13 ^b^
BSD11	5.05 ± 0.30 ^g^	1.97 ± 0.47 ^f,g^	38.21 ± 1.96 ^g^
BSD12	6.41 ± 1.33 ^b,c,d^	2.07 ± 0.27 ^e,f^	37.42 ± 1.29 ^g^
BSD13	7.03 ± 0.98 ^a^	2.17 ± 0.10 ^c,d,e^	37.46 ± 0.61 ^g^

TD, Traditional Drying; SD, Swell Drying; BTD, DIC Blanching + Traditional Drying; BSD, DIC Blanching + Swell Drying. ^1^ TPC = mg Gallic acid eq./g d.b. ^2^ TFC = mg Rutin eq./g d.b. ^3^ BC = μg betanin eq./g d.b. Values are expressed as means of two independent experiments in triplicate (*n* = 6). Letters in the same column indicate significative differences; Tukey HSD, *p* < 0.05.

**Table 2:** Trolox Equivalent Antioxidant Capacity (TEAC) and Free Radical Scavenging Activity by DPPH (IC_50_) of red dried beetroots.

Treatment	TEAC ^1^	IC_50_^2^
TD	278.03 ± 2.81 ^b^	2.69 ± 0.27 ^a^
SD	202.06 ± 13.49 ^h,i^	1.38 ± 0.03 ^h,i^
BTD	182.19 ± 9.30 ^j^	1.40 ± 0.06 ^g,h^
BSD1	225.11 ± 3.44 ^f,g^	1.90 ± 0.13 ^c,d^
BSD2	243.17 ± 1.49 ^c,d^	2.17 ± 0.14 ^b^
BSD3	298.72 ± 12.45 ^a^	2.02 ± 0.04 ^b,c^
BSD4	305.67 ± 5.75 ^a^	1.90 ± 0.17 ^c,d^
BSD5	222.61 ± 2.97 ^f,g^	1.52 ± 0.04 ^g,h^
BSD6	190.11 ± 4.52 ^i,j^	1.32 ± 0.07 ^h,i^
BSD7	229.97 ± 4.49 ^e,f^	1.20 ± 0.01 ^i^
BSD8	234.56 ± 13.31 ^d,e^	1.76 ± 0.09 ^e,f^
BSD9	260.67 ± 19.32^c^	1.32 ± 0.43 ^h,i^
BSD10	267.06 ± 12.72 ^b,c^	0.84 ± 0.01 ^j^
BSD11	255.25 ± 4.17 ^c,d^	0.99 ± 0.02 ^j^
BSD12	211.64 ± 11.99 ^g,h^	1.35 ± 0.06 ^h,i^
BSD13	205.94 ± 4.68 ^h^	1.62 ± 0.16 ^f,g^

TD, Traditional Drying; SD, Swell Drying; BTD, DIC Blanching + Traditional Drying; BSD, DIC Blanching + Swell Drying. ^1^ TEAC = (μM eq. of Trolox). ^2^ IC_50_ = (mg eq. gallic acid/g d.b). Values are expressed as means of two independent experiments in triplicate (*n* = 6). Letters in the same column indicate significative differences; Tukey HSD, *p* < 0.05.

**Table 3:** The experimental design for BSD red beetroots.

Blanched Swell Drying Red Beetroots (BSD)													
Run	1	2	3	4	5	6	7	8	9	10	11	12	13
Pressure, P (MPa)	0.35	0.60	0.35	0.35	0.53	0.53	0.35	0.17	0.17	0.35	0.10	0.35	0.35
Time, t (s)	20	20	35	20	31	9	20	9	31	20	20	5	20
